# Risk factors for the presence of *Fasciola hepatica* antibodies in bulk-milk samples and their association with milk production decreases, in Cuban dairy cattle

**DOI:** 10.1186/s12917-018-1654-2

**Published:** 2018-11-08

**Authors:** Amilcar Arenal, Yipsi García, Lídice Quesada, Dayamis Velázquez, Diamela Sánchez, Mayelin Peña, Asnaldo Suárez, Arnielis Díaz, Yuliet Sánchez, Stijn Casaert, Jan van Dijk, Jozef Vercruysse, Johannes Charlier

**Affiliations:** 1 Department of Morpho-Physiology, University of Camagüey Ignacio Agramonte Loynaz, Km 5 ½, 74650 Camagüey, Cuba; 2 Department of Virology, Parasitology and Immunology, Faculty of Veterinary Medicine, Ghent University, Salisburylaan 133, 9820 Merelbeke, Belgium; 3 Centre for Preventive Medicine, Animal Health Trust, Lanwades Park, Kentford, Newmarket, Suffolk, CB8 7UU UK; 4 Kreavet, Hendrik Mertensstraat 17, 9150, Kruibeke, Belgium

**Keywords:** Bulk-tank, Milk production, Risk factors, Liver fluke, Helminth, Ruminants, ELISA

## Abstract

**Background:**

Worldwide, *Fasciola hepatica* infection causes high production losses in the livestock industry. Recently, studies have analyzed the association between measurements of *F. hepatica* infection intensity and herd management practices. The aim of the present study, the first of its kind in a subtropical region, was to evaluate associations between *F. hepatica* bulk-tank milk ELISA results with herd management factors and milk yield in dairy herds, in Camagüey, Cuba. The SVANOVIR® *F. hepatica*-AB ELISA was used to measure *F. hepatica* antibody levels in a random sample of 516 dairy herds during the period of May–July of 2014. Farm management practice data were collected using a questionnaire.

**Results:**

With 82% of the herds testing positive, the results indicate that *F. hepatica* is very widespread in this area. Reductions in milk production of 18 and 32% were observed in herds with Optical Density Ratios (ODR) of 0.3–0.6 and > 0.6, respectively, when compared to herds with ODR <  0.3. Overall, the longer the milking cows were put out to pasture, the higher the levels of anti-parasite antibodies. Co-grazing with sheep and goats also significantly increased the risk of high ODR.

**Conclusions:**

Our data show a widespread occurrence of the parasite as well as a major potential impact of the infection on the Cuban development goal of becoming self-sufficient in milk production. Our risk factor analysis suggests that the prevention of infection around water sources, and the separation of cattle from small ruminants could be useful control measures. This is the first epidemiological survey of *F. hepatica* abundance, and associated reductions in milk yield, in dairy herds in Cuba.

## Background

During the 1980s, Cuban dairy cattle production levels enjoyed the highest growth in Latin America. In 1989, production peaked at 1134 million liters of milk. Since then, the Cuban dairy industry has faced momentous changes and challenges. During the Cuban economic crisis of the Nineties, milk production dropped back and was recorded at 353 million liters in 2005 [1]. At the same time, pure-bred Holstein herds, which had made up 72% of all herds, were reduced to 12%, with crossbreeding of Holstein dairy cows and Zebu cattle becoming the norm [2]. The percentage of dairy cows kept on privately-owned, as opposed to state-owned, farms increased from 20 to 80%. In recent years, milk production rebounded to approximately 600 million liters. However, this is estimated to be only 50% of the current Cuban milk demand [2]. To raise self-sufficiency levels in subtropical countries like Cuba, it is clearly important to evaluate existing milk production limitations.

In Cuba, milk production is based on the utilization of pastures in the rainy season and green and preserved forages, supplemented by sugar-industry by-products, in the dry season. The most important limitation on milk production in Cuba is thought to be that these nutritional resources contain less than desirable energy density [3]. This lack manifests itself especially in the dry season, by halving of milk production. Another likely factor reducing yield milk is infections, especially with helminths. However, the prevalence of economically important helminths on dairy farms, and their impact on milk production, has not been quantified in Cuba. Existing impact studies were all carried out in different climatic zones, and for very different farming systems [4, 5], and therefore it is unlikely that the results of such studies can be applied to milk production systems in subtropical regions.

Helminth infections are recognized as a major limitation for livestock production throughout the tropics and elsewhere [6]. Among these, infections with *Fasciola hepatica* are responsible for significant economic losses in the cattle industry, due to mortality, reduced production of meat and milk and costs of deworming.

Various diagnostic methods based on detecting antibodies specific for *F. hepatica* in feces, serum, meat juice and milk have been described previously [7–9]. The wide availability and simplicity of these tests have facilitated large epidemiological studies [10] and evaluation of the association between fluke infection status and milk production parameters [11]. The magnitude of such effects has been shown to depend on the production system [4, 12, 13], lending argument to the need to study such losses in disparate epidemiological and production settings.

To date, the only epidemiological data available in Cuba are prevalence data from routine inspections in slaughterhouses in the central provinces showing prevalences of 20–50% for *F. hepatica* [14, 15]. To define the potential constraint of helminth infections on dairy productivity and initiate the development of *F. hepatica* herd management recommendations, we conducted a targeted survey in the major milk producing province of Camagüey and deployed a bulk-tank milk (BTM) ELISA test as a tool for diagnosis of fasciolosis in Cuban dairy cattle.

## Results

### *Fasciola hepatica* antibodies

The mean, SD and range of the *F. hepatica* ODR were 0.510, 0.201 and 0.049 to 1.192, respectively. According to the manufacturer’s interpretation criteria 82.2% of the herds tested positive for *F. hepatica* (> 0.3 ODR, 95% confidence interval: 0.561–0.591), while 35.7% of herds were likely to suffer significant production decreases (> 0.6 ODR, 95% confidence interval: 0.705–0.736).

### Associations of *Fasciola hepatica* antibodies and management factors with milk yield

**Fig. 1:**
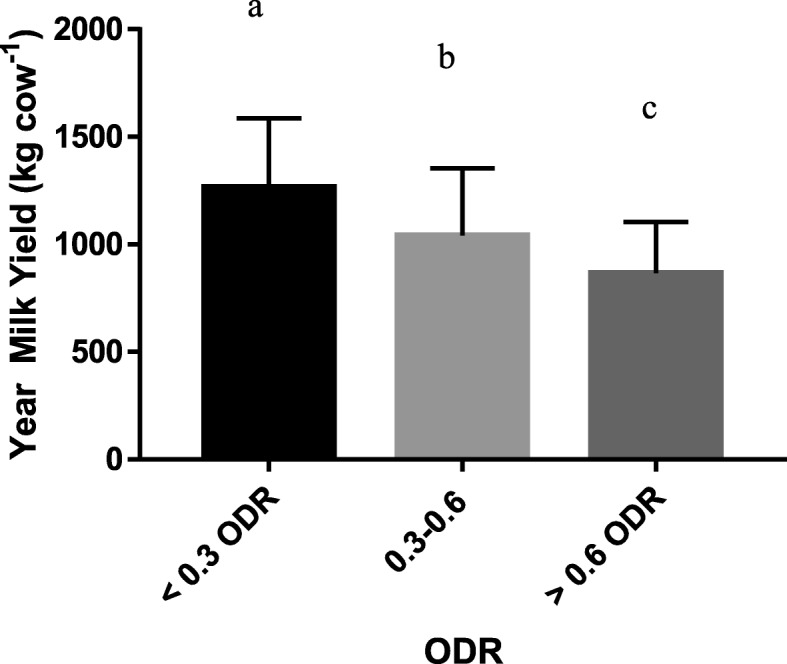
Milk production per cow per year according to the ELISA cut-off of the relative optical density ratio (ODR). Data are presented as mean ± SE (< 0.300, *N* = 92; 0.300–0.600, *N* = 257; > 0.600, *N* = 167). Bar indicates the SE. Different letters indicate significant differences among groups (*P* <  0.001)

Complete data (consisting of BTM ELISA results combined with complete questionnaire and milk production information) was obtained from 516 out of the 650 selected farms. The observed average milk yield per dairy cow per year was 1024 kg (95% confidence interval: 996–1051 kg). The average milk yield per dairy cow per year of the negative herds (< 0.3 ODR) was 1266 kg (95% confidence interval: 1200–1333 kg). There was a significant negative correlation between ODR and milk yield (*R* = − 0.44; *P* <  0.01). In the one-way ANOVA, the differences in average milk yield per cow per year between *F. hepatica* > 0.6 ODR and 0.3–0.6 ODR comparing to negative herds (< 0.3 ODR) were 401 kg (32%) and 226 kg (18%), respectively (Fig. 1).

The management factors that were significantly associated through univariable regression with milk yield are listed in Table 1. Grass proportion in the dry season (higher milk yield with higher grass proportion), watering place (lower milk yield for pool/pond/brook vs. well), farm total area (lower milk yield in smaller farms), municipality, nutritional supplement in dry (lower milk yield in case of supplement) and rainy season (higher milk yield in case of supplement), and ODR (lower milk yield with higher ODR) were all associated with milk yield. The multivariable regression model to investigate the association between ODR and milk yield (Table 2) retained ODR and municipality as the two significant predictors. According to this model, an increase in *F. hepatica* ODR over the interquartile range (0.33–0.64) is associated with a reduction in average milk yield of 183 kg/cow per year (14%).

**Table 1:** Variables significantly (*P* <  0.05) associated through univariable regression with annual milk yield (kg/cow/year) in Camagüey province, Cuba

Variable	Parameter	B	Std. Error	R^2^	Sig.
ODR	Intercept	1387.5	34.8	0.197	
	ODR	− 712.3	63.5		< 0.001
Municipality^a^	Intercept	978.1	34.9	0.153	
	Municipality				< 0.001
Grass proportion^a^	Intercept	940.7	25.8	0.051	
	Grass proportion				< 0.001
Watering place ^a^	Intercept	1044.0	15.9	0.015	
	Watering place				0.006
Farm total area^a^	Intercept	1050.6	24.7	0.012	
	Farm total area				0.048
Nutritional supplement in dry season^a^	Intercept	1054.7	16.8	0.022	
	Nutritional supplement dry season				0.001
Nutritional supplement in rainy season^a^	Intercept	945.3	21.9	0.040	
	Nutritional supplement in rainy season				< 0.001

^a^Different levels not shown

**Table 2:** Association between *F. hepatica* antibody level and milk yield (kg/cow per year) in Camagüey province, Cuba^a^

Parameter	B	Std. Error	Sig.
Intercept	1300.3	49.4	< 0.001
ODR	− 590.9	68.1	< 0.001
Municipality			< 0.001
Camagüey	− 107.0	50.3	0.034
Céspedes	− 138.1	81.9	0.093
Esmeralda	−102.2	72.2	0.157
Florida	−100.2	66.9	0.135
Guaimaro	129.7	46.5	0.005
Jimaguayú	16.8	50.5	0.739
Minas	−35.1	66.9	0.600
Najasa	86.5	54.5	0.113
Nuevitas	13.3	67.0	0.842
S.Cubitas	64.9	62.7	0.302
Santa Cruz del Sur	144.8	54.6	0.008
Sibanicú	70.2	55.8	0.210
Vertientes	Base	.	.

^a^Multivariable linear regression model (*R*^*2*^ = 0.264, *N* = 516 dairy herds)

### Associations of *Fasciola hepatica* antibodies with management factors

The frequencies at which different categories of management variables were measured, and their corresponding ODR are listed in Table 3. The UEB farms (state sector) presented significantly higher ODR values compared to the private sector (CPA and CCS) and also UBPC (state sector). Farms with more than 30 ha had a significantly higher ODR compared to the smaller ones. When the cows were grazed with sheep and goats the ODR was significantly higher compared to the farms where cows are grazed without other species or only with horses. Herds with access to pool/pond/brookhad significantly higher ODR compared to herds with wells as water source. It was also observed that farms with a lower grass proportion in the cow’s diet in the dry season presented a significantly higher ODR. In the dry season, a lower ODR value was also found when the grazing time was less than 6 h. The multivariate regression model identified grass proportion in the dry season and municipality as the two most significant associated factors with *F. hepatica* ODR (Table 4).

**Table 3:** Frequency and percentages of the herd characteristics of dairy herds sampled in Cuba (*N* = 516) in a cross-sectional questionnaire survey conducted in March–July, 2014

Variable			%	N	Mean ODR	SD	Range
Type of production	State^a^	UEB	10.1	52	0.600^a^	0.188	0.05-0.99
		UBPC	25.6	132	0.522^b^	0.187	0.05–0.98
	Private^a^	CPA	15.1	78	0.496^b^	0.177	0.07–0.89
		CCS	49.2	254	0.490^b^	0.212	0.05–1.19
Type of Herd	Dairy only		54.8	283	0.511 ^a^	0.208	0.05–1.19
	Both beef and dairy		45.2	233	0.509 ^a^	0.192	0.05–1.03
Farm total area	<=13.42 ha		32.8	169	0.474^b^	0.194	0.12–1.03
	> 13.42 and < =30 ha		14.0	72	0.472^b^	0.186	0.05–0.88
	> 30 ha		53.3	275	0.543^a^	0.204	0.05-1.19
Herd size (adult cows: lactating + dry):	< 30		55.4	286	0.499^a^	0.217	0.05-1.03
	30–60		30.6	158	0.539^a^	0.200	0.05-1.19
	> 60		14.0	72	0.534^a^	0.215	0.07-0.89
Deworming of cows			3.1	16	0.539^a^	0.187	0.28-0.98
Not dewormed							
Dewormed when worm problems			22.9	118	0.480^a^	0.197	0.05-0.89
Preventive treatment			74.0	382	0.518^a^	0.202	0.05-1.19
Cows grazed together with other species							
Sheep and goats			19.2	99	0.552^a^	0.178	0.05-1.03
Horse			21.5	111	0.501^b^	0.196	0.12–0.99
None			59.3	306	0.500^b^	0.208	0.05–1.19
Stocking rate: average number of cows per hectare on a parcel?							
< 1			40.3	208	0.496^a^	0.185	0.07-1.19
1–2			56.2	290	0.521^a^	0.210	0.05-0.99
> 2			3.5	18	0.501^a^	0.232	0.05-0.85
Watering place							
Pool/pond/brook			20.9	108	0.561^a^	0.206	0.07-1.19
Pump on pasture			79.1	408	0.497^b^	0.198	0.05–0.99
Rotational grazing of cows							
Yes			5.4	28	0.511^a^	0.190	0.15-0.82
No			94.6	488	0.502^a^	0.201	0.05-1.19
What was the cow’s grazing time per day during the dry season?							
Day and night			14.0	72	0.542^a^	0.165	0.05-0.76
< 6 h per day			62.6	322	0.498^b^	0.217	0.05–1.19
> 6 h per day			23.4	122	0.541^a^	0.167	0.05-0.98
What was the cow’s grazing time per day during the rainy season?							
Day and night			11.0	57	0.513^a^	0.168	0.05-0.80
< 6 h per day			42.4	219	0.504^a^	0.205	0.05-1.19
> 6 h per day			46.5	240	0.515^a^	0.205	0.05-0.99
Grass proportion dry season							
81–100			20.5	106	0.406^c^	0.201	0.05–0.88
51–80			50.6	261	0.511^b^	0.198	0.07–0.99
< =50			28.9	149	0.583^a^	0.172	0.05-1.19
Grass proportion rainy season							
81–100			30.4	157	0.501^a^	0.188	90.05-0.91
51–80			52.3	270	0.519^a^	0.205	0.05-1.19
< =50			17.2	89	0.501^a^	0.215	0.12-1.03
Grass Mowing							
51–100%			27.9	144	0.496^a^	0.187	0.05-0.88
< 50%			43.0	222	0.512^a^	0.210	0.05-1.03
Never			29.1	150	0.522^a^	0.200	0.07-1.19
Municipality	Camagüey		10.7%	55	0.630^a^	0.114	0.37-.87
	Céspedes		2.7%	14	0.634 ^a^	0.303	0.05–1.19
	Esmeralda		3.7%	19	0.493 ^a,b,c^	0.196	.15–.82
	Florida		4.5%	23	0.548 ^a,b^	0.171	0.25–.86
	Guaimaro		15.7%	81	0.381 ^c,d^	0.179	0.05–.88
	Jimaguayú		10.3%	53	0.567 ^a,b^	0.179	0.07–.99
	Minas		4.5%	23	0.511 ^a,b^	0.254	0.09–.88
	Najasa		8.1%	42	0.455 ^b,c,d^	0.142	0.17–.77
	Nuevitas		4.5%	23	0.598^a,b^	0.263	0.07–.99
	S.Cubitas		5.6%	29	0.360 ^d^	0.192	0.11–.91
	Santa Cruz del Sur		7.9%	41	0.572 ^a,b^	0.177	0.15–.91
	Sibanicú		7.6%	39	0.464 ^b,c,d^	0.171	0.17.84
	Vertientes		14.3%	74	0.545 ^a,b^	0.179	0.05.88

Different letters indicate significant differences between groups (*P* <  0.05)

^a^UEB (Basic unit from the state), UBPC (Cooperative Unit Basic of Production), CCS (Credit and Service Cooperative) and CPA (Agropecuary Production Cooperative)

**Table 4:** Multivariable linear regression model of management factors associated with *F. hepatica* ODR measured in bulk-tank milk samples in Camagüey province, Cuba^a^

Parameter	B	Std. Error	Sig.
Intercept	0.579	0.025	< 0.001
Grass proportion dry season (%)			0.004
81–100	− 0.110	0.033	0.001
50–80	−0.022	0.022	0.317
< 50	Base		
Municipality			< 0.001
Camagüey	0.070	0.033	0.033
Céspedes	0.060	0.054	0.268
Esmeralda	−0.062	0.048	0.197
Florida	−0.010	0.044	0.827
Guaimaro	−0.099	0.035	0.005
Jimaguayú	0.009	0.033	0.791
Minas	−0.046	0.046	0.308
Najasa	−0.112	0.036	0.002
Nuevitas	0.040	0.046	0.374
S.Cubitas	−0.198	0.042	0.000
Santa Cruz del Sur	0.006	0.036	0.874
Sibanicú	−0.093	0.038	0.015
Vertientes	Base	.	.

^a^(*R*^*2*^ = 0.204, *N* = 516 dairy herds)

## Discussion

Our study found evidence of *F. hepatica* infection on 4 out of 5 farms in the major milk producing province in Cuba. Moreover, this infection was not only highly prevalent, but also significantly associated with decreases in milk yield.

In previous abattoir-based studies in Cuba, *F. hepatica* parasites were observed to be present between 20 and 50% of the animals. However, the latter studies were conducted at the individual cow level [14, 15] and in a different geographical region of Cuba. Moreover, it is known that meat inspection at the slaughterhouse has a lower sensitivity than serology-based methods [16].

The evidence of widespread *F. hepatica* infection in Cuban dairy herds, together with the known deleterious effects of *F. hepatica* on animal welfare and productivity suggest than these infections should be considered of major importance in Cuban dairy farms. However, on high-input, intensive, Holstein-pedigree farms, milk production per cow per year was more than 6 times that of the Cuban mixed breed cattle studied here. Cuban cattle are not genetically capable of achieving such high levels of milk production, they eat a less nutritious diet and, in subtropical climates, they often face higher parasite burdens. Because Cuban cattle are likely under lower metabolic stress than their European counterparts in intensive production systems, it could be proposed that parasite-ascribed decreases in milk production in Cuba should be lower than, for example, in Europe. However, in the UK, in high yielding herds, *F. hepatica* – associated decreases were estimated at 15% [4], compared to estimated decreases of 18% to 32% in the present study. The Cuban estimate is substantially higher than the 3% reduction described in Belgium in herds with high ODR [11] and the 6% in Spanish herds with high infection levels [13]. Partly, these differences may be ascribed to the fact that we did not control for some confounding factors, such as lactation stage, age composition or somatic cell count data in our analysis. This was not possible, as these data are not routinely collected in Cuban dairy farms. Therefore, further elucidation of the true and recoverable production impact would require an intervention trial using anthelmintic treatment under field conditions [17].

The impact of parasite infections on food security may be more keenly felt in countries where demand is already outstripping supply. At the same time, options for control are likely to be more limited in subtropical systems. For example, with very few water sources available, options for pasture rotation are limited. In Cuba, anthelmintic treatments for *F. hepatica* are not used routinely either because of a lack of availability in the Cuban market and/or a lack of diagnostic routine. This study made a start with the identification of risk factors, which should aid in the development of control recommendations for the Cuban dairy sector. Different farming systems had different ODR levels. UEB farms, which are normally the larger farms, with more extensive access to suitable habitats for lymnaeid snails, had higher ODRs. Similarly, farms with a higher number of hectares available had higher ODRs. In Denmark, larger dairy herds were also more prone to *F. hepatica* infection [18]. In Turkey and Tanzania, large-scale and traditional (stationary herds without effective disease control) dairy farms presented higher prevalence of *Fasciola* sp*.* than small-scale farms [19, 20]. This may be related to intensively grazed pastures and to an increased likelihood of cattle encountering fluke-contaminated snail habitats on larger farms.

Access to suitable habitats for lymnaeid snails, usually man-made ponds of stagnant water used to water cattle, indeed appears to be an important factor contributing to higher levels of infection. In this study, farms with less grass as a proportion of the total diet, available during the dry season, had significantly higher antibody titers. On these farms, cattle will normally be congregated around these habitats for lymnaeid snails and receive supplementation with other food sources, such as sugar cane byproducts. They will therefore have increased contact time with metacercaria-contaminated snail-infested areas. Access to, and type of, water sources could be the key overriding factor in fluke transmission in Cuba. This may be an important area to focus on in terms of limiting losses to the parasite.

Grazing alongside horses was not a significant risk factor whereas, in agreement with other studies [21], co-grazing with small ruminants clearly increased the risk of higher ODR levels.

There were significant differences in ODR levels between municipalities. The reason for this could include local environmental differences as well as differences in local farm management practices [22]. In the UK, McCann, Baylis and Williams [23] detected rainfall as the main responsible factor of variation (23%) in *F. hepatica* BTM antibody levels, whilst farm management explained about 21% of variation. Bennema et al. [24] found that in regions with relatively homogenous climatic and environmental conditions, management factors are the primary factors determining *F. hepatica* infection risk. Further research is recommended to determine the importance of water source as well as of infection and the local environmental (soil type, local pasture, infection with other parasite, bacterial interactions, landscape features) and climatic conditions affecting the infection risk. Moreover, it is necessary to evaluate the impact of host factors such as age and genetic make-up. Ultimately, this could result in local risk maps and evidence-based and practical management recommendations such as sanitation of pastures and water sources and targeted anthelmintic treatment during periods of highest infection pressure [24, 25].

## Conclusions

We have provided baseline *F. hepatica* exposure data for the major milk production area of Cuba. Our data show a widespread occurrence of the parasite, as well as a major potential impact of this infection on the Cuban development goal to become self-sufficient in milk production. Our risk factor analysis suggests that the prevention of infection around habitats suitable for lymnaeid snails, and that the separation of cattle and small ruminants could be useful control recommendations. However, further research to confirm the importance of these risk factors as well as to understand the basic *F. hepatica* epidemiology in relation to temporal and regional changes in climate and landscape in Cuba is needed.

## Methods

### Study area

The study was conducted in Camagüey province, eastern Cuba. Camagüey has a surface of 15,615 km^2^ and a tropical climate with an average annual temperature and rainfall of 24.7 °C and 1200 mm (www.one.cu), respectively. Elevation varies slightly, from sea level at the coast to 100 m in the center. According to the milk industry department of the Ministry of Agriculture, in Camagüey, approximately 10,000 dairy farms provide milk to a dairy cooperative during the rainy season (March–July); however, during the dry season (August–February) the number of dairy farms providing milk dropped to below 6000 (Reynaldo González, personal communication).

### Sampling and laboratory procedure

The farms were selected based on the following criteria: (a) storage of farm production data in the milk industry department of the Ministry of Agriculture, in Camagüey; (b) providing milk during the whole year; (c) proportionally according to the total farm per municipality and (d) farmers agree to participate. Using the RANDBETWEEN function in Microsoft® Excel, 650 BTM samples were randomly chosen out of all available regional samples (N ≈ 6000). We collected the samples during the period of May–July 2014. The 650 dairy farms were located across the 13 municipalities in Camagüey. We transported the samples to the laboratory within 4 h after collection. The samples were kept at 4 °C between collections on the farms. After arrival at the laboratory, the milk samples were centrifuged (16,000 × *g*, 5 min), fat was skimmed off and the supernatant was collected and frozen at − 20 °C (for a maximum period of three months) until further analysis.

Samples were analyzed using a commercially available ELISA test (SVANOVIR® *F. hepatica*-Ab, Svanova Biotech, Uppsala) according the manufacturer’s instructions. The ELISA results are expressed as optical density ratios (ODR). ODR = (OD - NC) / (PC - NC), where OD is the optical density at 405 nm of the sample and NC and PC are the OD at 405 nm of the negative and positive controls, respectively.

### Questionnaire

We collected the management data by interviewing the farmers in person. Information was collected on location, herd size, type of production (private or state), watering place [pool/pond/brook or well], pasture management and other husbandry practices, and anthelmintic control measures in adult cows. A complete list of the collected variables is provided in Table 1. Milk production data were collected from the milk industry department of the Ministry of Agriculture.

### Associations between *F. hepatica* antibody levels and milk yield

The association between *F. hepatica* BTM antibody level (ODR) and the average milk yield per cow per year (referred to as “milk yield”) was first investigated by the Pearson correlation coefficient. Next, milk yield was compared by a one-way ANOVA with a Student–Newman–Keuls multiple comparisons post-hoc test between the quartiles of the *F. hepatica* ODR. In addition, one-way ANOVA, was also used to evaluate the association of farm management factors with milk yield, for each management factor independently. Finally, a multivariable model was built to assess the association of *F. hepatica* ODR with farm management factors (=independent variables) and milk yield (= outcome variable).

### Associations between *F. hepatica* antibody levels and management variables

First, a one-way ANOVA was used to test for significant differences in ODR between the different categories of each farm management factor. The differences were further analyzed using the Student–Newman–Keuls multiple comparisons post-hoc test. For comparisons of factors with only two categories, the Student t-test was used.

Factors that were significant in this first screening (α = 0.05) were evaluated in a multivariable linear regression model that was constructed by forward stepwise selection of variables with a nominal significance level of α = 0.05 and 0.10 for the entry and removal of a variable, respectively. Two-way interactions between the variables included in the final model were evaluated for significance. In all the above models, a level of α = 0.05 was used to declare a variable to be statistically significant. The analysis was conducted in SPSS v21.0 (SPSS Inc., Chicago, USA).

## Abbreviations

BTM: Bulk tank milk
CCS: (Cooperativa de Crédito y Servicio) Credit and Service Cooperative
CPA: (Cooperativa de Producción Agropecuaria) Agricultural Production Cooperative
ODR: Optical Density Ratios
UBPC: (Unidades Básicas de Producción Cooperativa) Basic Units of Cooperative Production (The new farms which now make up the largest sector in Cuban agriculture)
UEB: (Unidades Empresariales de Base) State-owned Basic Business Units
